# Computerized Neuropsychological Assessment in Aging: Testing Efficacy and Clinical Ecology of Different Interfaces

**DOI:** 10.1155/2014/804723

**Published:** 2014-07-24

**Authors:** Matteo Canini, Petronilla Battista, Pasquale Anthony Della Rosa, Eleonora Catricalà, Christian Salvatore, Maria Carla Gilardi, Isabella Castiglioni

**Affiliations:** ^1^Institute of Molecular Bioimaging and Physiology, National Research Council (IBFM-CNR), Segrate, 20090 Milan, Italy; ^2^Laboratory of Neuropsychology, The Foundation of the Carlo Besta Neurological Institute, IRCSS, 20133 Milan, Italy

## Abstract

Digital technologies have opened new opportunities for psychological testing, allowing new computerized testing tools to be developed and/or paper and pencil testing tools to be translated to new computerized devices. The question that rises is whether these implementations may introduce some technology-specific effects to be considered in neuropsychological evaluations. Two core aspects have been investigated in this work: the efficacy of tests and the clinical ecology of their administration (the ability to measure real-world test performance), specifically (1) the testing efficacy of a computerized test when response to stimuli is measured using a touch-screen compared to a conventional mouse-control response device; (2) the testing efficacy of a computerized test with respect to different input modalities (visual versus verbal); and (3) the ecology of two computerized assessment modalities (touch-screen and mouse-control), including preference measurements of participants. Our results suggest that (1) touch-screen devices are suitable for administering experimental tasks requiring precise timings for detection, (2) intrinsic nature of neuropsychological tests should always be respected in terms of stimuli presentation when translated to new digitalized environment, and (3) touch-screen devices result in ecological instruments being proposed for the computerized administration of neuropsychological tests with a high level of preference from elderly people.

## 1. Introduction

Computerized neuropsychological tests have been used in research for almost fifty years [1]. Although many different test batteries have been developed and new batteries are introduced every year for clinical screening, not sufficient normative data and standardized psychometric measures are yet available [2]. Conversely, paper and pencil tests are widely approved and are still regarded as keynote tools for neuropsychological assessment, due to their high validity and reliability [3]. Paper and pencil neuropsychological tests are based on the presence of a neuropsychologist, essential for the assessment of cognitive abilities, especially for the evaluation of a person with brain injury or cognitive impairment and for the selection, administration, and interpretation of tests. Although suffering from some levels of subjectivity, variability, and long times (due to the fact that it is often necessary to do a screening and also a diagnostic deepening), paper and pencil tests have been validated for the administration of reliable tests able to pinpoint a potential deficit involving a specific cognitive ability, or to discriminate among impairments in different cognitive domains [4]. However, neuropsychological evaluation can also provide information concerning normal brain functioning and allows monitoring the cognitive status of an individual, especially throughout older age. Therefore, its results are extremely important to trace a continuum of normal functioning in the aging population, not only in presence of pathologies. Documenting changes in cognition is, indeed, an important issue in neuropsychological assessment, as the clinician/researcher is often called upon to determine if and when cognitive functioning has changed. Another important advantage of the conventional paper and pencil neuropsychological assessment is their ecological validity. In the context of neuropsychological testing, ecological validity refers to the degree to which test performance corresponds to real-world performance. Validity does not apply to the test itself, but to the inferences that are drawn from the test [5, 6]. Most importantly, clinical sensitivity allowing neuropsychologist to capture potential shades in a specific domain and to trace a specific cognitive profile may results difficult to be translated in a computer-based assessment. Despite these numerous advantages, traditional paper and pencil tools show some limitations particularly when assessing cognitive changes in a relatively short follow-up period. The most commonly administered tests usually do not provide alternative forms of administration [7], thus precluding to repeat testing over short intervals (e.g., <6 months) [8]. Other specific limitations concern the intrinsic nature of the tools and include: assisting the setting and manually computing scoring by the experimenter [9], long duration of the assessment, potential bias related to different examiners [10], impossibility to provide a precise time control on stimuli presentation and/or the lack of an accurate measurement of motor response accuracy [11], and greater costs. Although there are specific tests for the assessment of attentional and executive functions which can evaluate individual components and can diagnose specific deficits [12], some executive/attention abilities could take advantage from the use of a computerized tool, in particular in the assessment of response inhibition, resistance to distraction, planning, problem solving, working memory operations, and mental set shifting divided attention.

All of these limitations could be overcome by a computerized assessment, on condition that efficacies and ecological measurements are carried out. About these issues, the American Psychological Association (APA) has recognized the importance of computerized psychological testing and has suggested how to implement and interpret computerized test results in its guidelines [13]. Furthermore, computerized assessment of cognitive functions can be self-administered and can have a shorter duration (e.g., by reducing “dead” times in stimuli presentation). They may have great validity and reliability due to their great objectivity, precision, and standardization. Computerized performance can also minimize the so called “floor and ceiling effects”, occurring when differences among participant performance are not fully captured; thus, they can provide more standardized measures of subject performance, crucial for example, for an accurate and early detection of specific pathological disease (e.g., dementia) [14].

It appears clearly that computerized testing will represent an essential part of the clinical setting in the nearest future, above all, in screening procedures, on condition that these new instruments and their results are governed by experts.

Feasible, efficacious, and ecological computerized testing could allow clear pictures of normal cognition to be measured and monitored also at home, pinpointing specific deficits in each cognitive domain in aging people. Validated computerized tools could also provide stronger grounding to overcome the lack of a consensus regarding the feasibility and testing efficacy related to the different types of technological solutions and settings and/or response layouts chosen for the assessment. For instance, a more ecological technological solution can overcome one important obstacle to the wide use of computerized assessment attributable to the familiarity with technological devices [11], while a more ecological setting in the nearest future could partially allow the administration of the tests without the support of a specialized clinician, or the tests could be potentially self-administered or assessed by a caregiver at home.

The aims of this study were to evaluate the following:

1. the testing efficacy of a computerized test (in a representative case of executive function assessment) when response to stimuli is measured using a touch screen compared to a conventional mouse-control response device;
2. the testing efficacy (the efficacy of the neuropsychological test) of a computerized test (in a representative case of memory function assessment) with respect to two different input modalities: a visual presentation modality of the test, replicating the most diffused digital versions of the test, and a verbal presentation modality, replicating the classical clinical administration of the test;
3. the ecology of both computerized assessment modalities (touch-screen tablet and mouse-control PC), including preference measurements of participants.

## 2. Related Works

Computerized cognitive batteries are already available and used for the screening and the assessment of dementia. Some of these instruments were appropriately created for the assessment of cognitive decline in dementia; other ones were adapted to fulfill this role in aging. Some tools are designed for research use, and others tools have been designed mainly for clinical use, some of them already implemented in clinical guidelines.

An interesting review [2] reported seventeen computerized test batteries used in the measurement of cognitive abilities of adults. Some of these tools are able to run only on a PC/laptop and others are available only on web sites.

Among these tools:

1. CNS vital signs [15] battery is developed as a routine clinical screening instrument. It includes seven tests: verbal and visual memory, finger tapping, symbol digit coding, Stroop test, test of shifting attention, and continuous performance test.
2. CogState [16] battery was developed as a dementia screening instrument and it is implemented as a card game form. The participant plays different games that are adapted accordingly to performance. CogState requires an active internet connection to generate a report. The participant's data are uploaded and analyzed. Then a report is generated and e-mailed back to the provider.
3. NeuroTrax [17] includes custom software on the local testing computer and serves as a platform for interactive cognitive tests that provide precise accuracy and reaction time data. The level of difficulty is graded. The NeuroTrax tests different cognitive domains, memory (verbal and nonverbal), executive function, visual spatial skills, verbal fluency, attention, information processing, and motor skills. The tests were designed for use with the older adults. Responses are collected with the mouse or with the number pad on the keyboard. NeuroTrax also generates a report containing raw and standardized scores immediately following testing. Administrative features are web based.
4. IntegNeuro [18], in brief, investigates the following domains of cognitive function: sensorimotor, verbal and language, memory, executive planning, and attention. Scoring of responses is obtained by using an automated software program. Trained research assistants conducted the hand scoring of some tests and oversight is implemented to monitor accuracy.
5. Touch panel dementia assessment scale [19] hardware comprises a 14-inch touch panel display and computer devices built into one case. The TDAS runs on Windows OS and was bundled with a custom program made with reference to the ADAS-cog with the elderly under the control of a physician.

More recently, screening tools for assessment of cognitive impairment able to run on an iPad tablet device have made their appearance as well. They can be downloaded and are self-administrable (see Table 1).

CADi [20] and CANTAB mobile [21] are the first two tools which exploited this technology. The CADi consists of 10 very brief tests; the purpose is to provide a mass screening in the Japanese population in a relatively short time and with overall cost substantially lower than paper and pencil based examination. The most important limit of this tool is the cultural background underlying the validation, as it is available only in Japanese language. Notwithstanding, there are 18 different language versions of the CANTAB mobile tool; it comprises focused screening tests able to investigate only episodic memory and learning abilities (PAL task).

## 3. Materials and Methods

### 3.1. Participants

A group of 38 healthy participants was recruited among the Italian elderly population (20 males; age range = 53 : 87; age mean = 64.474; standard deviation [SD] = ±8.462; education range = 5 : 19; education mean = 11,263; [SD] = ±4.131).

Participants with any history of neurological illnesses and a Mini mental state examination (MMSE) score lower than 26 were excluded from the study (MMSE range = 26 : 30; MMSE mean = 28,711; [SD] = ±1.183) (see Table 2). Informed consent was obtained from all participants.

### 3.2. Hardware Device

In order to compare the participant responses from neuropsychological assessment delivered through mouse-control PC and touch-screen tablet, we used an Asus T100T notebook PC (CPU: quad core Intel Atom processor, RAM: 2 GB, Screen: 10.1′′ HD, and screen resolution 1366∗768 with multitouch). This device consists of a 10.1 inches tablet running a Windows 8.1 OS which can be used as a standalone touch-screen device or, combined with a mobile dock, as a “standard” PC with conventional input peripherals (mouse and keyboard).

### 3.3. Software and Scripting

The experimental tasks were implemented and administered using the presentation software (http://www.neurobs.com/). This is an object-oriented programming language allowing a sharp control on stimuli presentation (e.g., objects presentation timing and randomization of trials) and on response tracking. Experimental paradigms are set up through scripts consisting of two logical components: (i) a Scenario Definition Language (SDL) where the objects of the task (i.e., stimuli organized with hierarchical levels of complexity) are defined along with their specific timing and response matching and a (ii) Presentation Control Language (PCL) where objects presentation parameters are controlled (e.g., possibility to present stimuli through loops, conditionals, and subroutines for trials randomization). An additional third component, the SDL header, is used to specify general parameters, that is, those values which will be used as default for the presentation of all stimuli unless a different specification is given in the stimulus definition (e.g., default font size, background color, stimuli duration and timing, logfiles specifications, and so forth).

Presentation tracks and stores information about stimuli administration timing and participant response behaviors (i.e., the response given by the subject) in terms of response classification and reaction times. Information is subsequently analyzed using* ad hoc* data processing procedures in order to assess participant performance.

Parameters to be measured for response behaviors of participants are defined by the experimenter and specified in terms of (i) input device/s used by the participant and (ii) response buttons, in GUI's dedicated response setting panel, associated to the selected device to be recalled in the SDL component. Contextually to our experimental setting, two “response layouts” (i.e., mouse-control or touch-screen) were configured in the following manner.

- A “mouse response layout” where the docking-station built-in trackpad mouse was specified as input device, and pressing one of the two mouse buttons was specified as response behavior; two distinct classes of responses were coded, each one associated to a specific button press (i.e., pressing the left and right mouse buttons). Each response class was recorded and directly matched with the stimulus target response in order to assess the responses accuracy.
- A “touch response layout” where the capacitive screen surface was specified as input device, and the screen press was specified as response behavior: in this case this was the only available class of response. The two (i.e., left and right) response classes were obtained through data after processing: *X* and *Y* coordinates were registered each time the subject gave a response to a target stimulus and the *X* coordinate was used to estimate whether the response occurred on the left or right portion of the screen; positive *X* corresponded to right touch screen press, and negative *X* corresponded to left touch screen press.

### 3.4. The Neuropsychological Tests

#### 3.4.1. The Attentional Networks Test (ANT)

The attentional network test (ANT) [22] is designed to test three different attentional networks, namely, the executive control and the alerting and the orienting components of attention.

**Figure 1:**
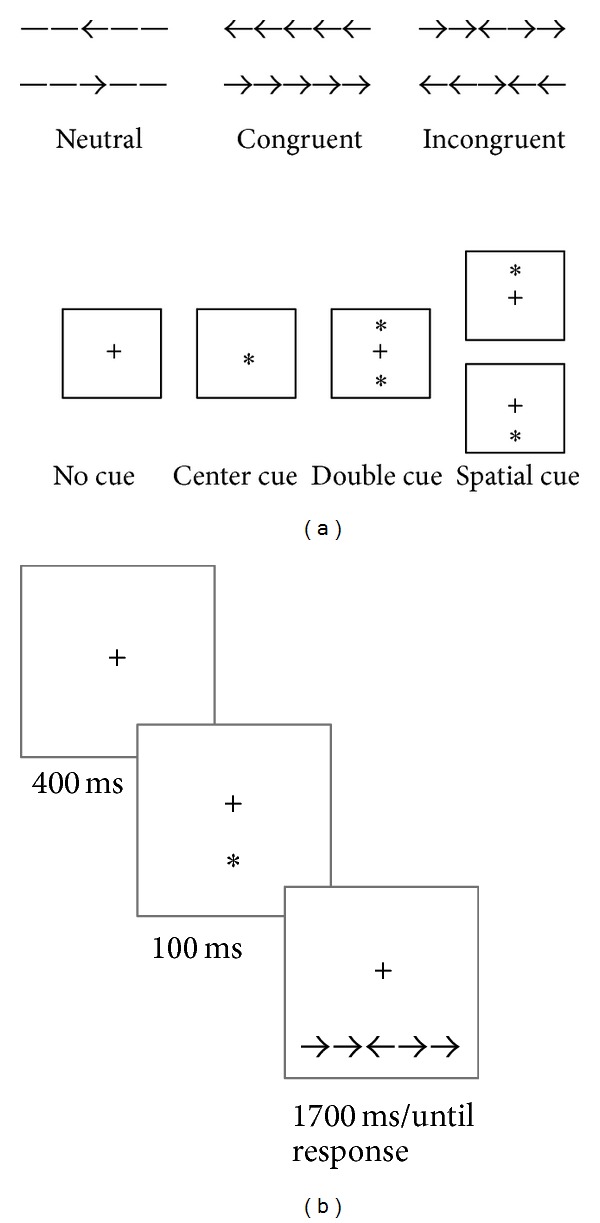
(a) Attentional network test experimental conditions are shown; each trial presented during the task is a combination of congruency (3 levels, on top) and cue (4 levels, below) conditions (adapted from [22]). (b) An example of, spatially cued, incongruent trial is presented; stimuli timings and interstimulus interval of this customized version of the task are provided below each stimulus (adapted from [23]).

Each trial starts with a fixation point and ends with the target stimulus, consisting of an array of five contiguous arrows; the participant is asked to state as fast as possible the direction (i.e., left or right) of the central arrow (see Figure 1).

The array can be defined according to a congruency factor (depending on the direction of the arrows flanking the central, target stimulus) and a spatial factor depending upon whether the array can occur either above or below the fixation point or at the center of the screen; further the presentation of the array can be cued or not by an asterisk (see Figure 1 for a detailed description of all experimental conditions). Different combinations of these experimental factors are selectively used to assess the efficiency of the three different networks (i.e., executive control and alerting and orienting component) through accuracy and reaction times (RT) analysis as follows.

- Conflict effect (CE) is assessed by subtracting RTs belonging to congruent trials (flanking arrows pointing in the same direction of the central target arrow) from RTs belonging to incongruent trials (flanking arrows pointing in the opposite direction of the central target arrow).
- Alerting effect (AE) is calculated by subtracting mean RT of the double cued trials (target trial presentation is preceded by two spatial cues occurring contemporarily above and below the fixation point) from the mean RT of the no cue trials (target trial is not preceded by any cue).
- Orienting effect (OE) is calculated by subtracting the mean RT of the central cue condition (target trial is preceded by a cue occurring at the center of the screen) from the mean RT belonging to the spatial cue condition (target trial is preceded by a spatial cue occurring either above or below the fixation point, depending on the position of the incoming target trial).

#### 3.4.2. The Auditory Verbal Learning Test (AVLT)

The Rey Auditory Verbal Learning Test [24] assesses the long-term verbal memory or the ability to learn and store in long-term memory unstructured verbal material. It consists of 5 consecutive repetitions for learning the material and then a long delay free recall 15 minutes later. The test consists of 15 unrelated words presented orally. First, the participant has to memorize this list of 15 words and immediately try to recall them after each presentation (immediate recall, IR). Second, after 15 minutes, in the meanwhile the participant is performing nonverbal tests, the participant is asked to recall the same list (delayed recall, DR). Finally, the subject is presented with a longer list of distracter words and asked to recall the previously learned words (recognition task). The Italian test has three alternatives but completely equivalent lists [25].

An interference task not involving verbal or memory cognition is administered between the third (i.e., last) IR session and the DR session.

### 3.5. Testing Efficacy I: Executive Function Assessment (ANT)

Given our purpose to compare the testing efficacy of a computerized test administered by touch-screen tablet versus conventional mouse-control PC, we created two versions of the ANT task: the first one for the* mouse layout response* and the second for the* touch layout response (see Section 3.3 Software and Scripting).*

Both versions were programmed using the presentation software and shared the exact same picture stimuli, stimuli timings, and randomization criteria; they were thus virtually equal, differing only for the input devices layout configuration.

#### 3.5.1. Mouse-Control PC Implementation

Each experimental condition of the ANT task was represented by 8 trials, resulting in a total of 96 [(3 congruency × 4 cue) × 8] trials for each experimental condition.

To this extent an array of 96 trials was specified, each trial consisting of a variable number of component stimuli, depending on the experimental condition.

Each trial was thus specified in terms of (i) stimuli objects, (ii) time intervals occurring between successive stimuli, and (iii) target response (see Figure 1).

The trial presentation order was specified using a fully randomized design. A set of three different interstimulus intervals (ISI) (min value 1873 ms, max value 4964 ms) was also specified; this means that a different time interval could occur between successive trials in order to avoid habituation effects. Two response buttons were assigned, and the participant was required to press as fast as possible the left or right mouse button (depending on the target trial type, as previously described).

Responses accuracy was coded in a logfile by comparing participant's response to a given target stimulus to the expected response for that stimulus, coded in the stimulus parameters in the SDL.

Stimuli presentation and response timing and accuracy were also recorded.

#### 3.5.2. Touch-Screen Tablet Implementation

A touch version of the ANT task was implemented using the same number of trials, experimental design, and randomization order of the ANT task in the* mouse*-control PC implementation. The only difference between the touch layout version and the mouse layout version resides in the response device assigned to the participant. While in the mouse version the participant was asked to respond by pressing the two mouse buttons, in this touch version of the task he/she was required to press the left most or right most part of the screen with the thumbs, handling the touch device with both the hands. Touch location was coded as previously described (e.g., negative *X* means a touch occurred in the left portion of the screen and corresponds to the left mouse button press).

### 3.6. Testing Efficacy II: Memory Function Assessment (AVLT)

Given our purpose to assess the efficacy of different modalities (i.e., verbal versus visual implementation) of a computerized test administered by a touch-screen tablet, two versions of the AVLT were created: (1) one verbal version (Verbal AVLT Task), consisting in the presentation of auditory stimuli and requiring the subjects to verbally* recall* answers (which were both recorded and coded by the tester), thus replicating the conventional administration of AVLT used in clinical context, and (2) one visual version (Visual AVLT Task), consisting in the presentation of visual list of words on the screen and requiring the subjects to* recognize* target stimuli by touching the screen when a presented target word appeared. We have chosen not to calibrate the level of difficulty for the recognition test stimuli to be equivalent to that of the recall stimuli in order to detect any differences between the two stimuli presentation modalities, including the level of difficulty.

#### 3.6.1. AVLT Equivalent Lists Creation

Since both lexical and psycholinguistic variables can influence behavioral performance, the characteristics of the original 15 Rey list words were extracted and analyzed in order to create 5 alternatives but completely equivalent lists. Words frequency values for the original 15 words were determined from the Italian lexicon [26], while for the familiarity (FAM), concreteness (CNC), age of acquisition (AoA), and imageability (IMG), values were extracted from the MoA database [27]. For each variable the mean of the distribution of values for the fifteen words and the interquartile range (75–25) was computed. Only the words falling in this range and with values for at least three variables overlapping with those of each word were selected. Following this criteria, 50 words were selected and matched to 10 original words included in the original Rey list. The 50 words were subsequently divided into 5 lists, each containing 10 words. Lists were balanced and statistically matched to the original Rey list. A one-way ANOVA analysis revealed no significant difference for (i) word length (*F*(5,54) = 0.095, *P* = 0.993), (ii) FAM (*F*(5,54) = 0.856, *P* = 0.517), (iii) CNC (*F*(5,54) = 1.146, *P* = 0.348), (iv) AoA (*F*(5,54) = 1,416, *P* = 0.233), and (v) IMG (*F*(5,54) = 1.096, *P* = 0.373). Bonferroni post hoc corrections for pairs of lists revealed no significant difference between lists for none of the above-mentioned variables (all *P* values <1). Semantic or phonemic similarities between words within each list were also excluded.

Among these five lists, two were selected as target stimuli lists, while words belonging to the remaining three lists were used as filler words for the recognition part of the visual version of AVLT (see Section 3.6.3). Each of the two-target lists was pseudorandomized according to three different word orders to control for possible word list sequence effects. Thus, one out of the resulting 6 lists (i.e., two words sets, each randomized three times) was selected as target list for each participant in both versions of the task.

#### 3.6.2. Verbal AVLT Task by the Touch-Screen Tablet

A list of 10 target words was presented verbally to the participants. Specifically, the words were administered as auditory signal, and the sound was generated using a text-to-speech software (Audacity, the free cross-platform sound editor software, http://www.audacity.sourceforge.net/), and recorded, refined and normalized in order to equalize all words in terms of voice quality and volume.

After each presentation the participant was asked to verbally recall as much words as possible.

The participant was tested three times consecutively as in [19] for the immediate memory recall and once for the delayed memory component. Vocal recordings of target words were used as stimuli in this task version and were administered through the integrated speakers of the touch screen tablet. Stimuli were presented consecutively with an interstimulus interval (ISI) of 3000 ms occurring between one stimulus and the next one. Recall sessions were tested using a voice recording script in order to store an audio logfile of participant responses. Parallel to the sound recording, the 10 target words were constantly displayed on the screen only for the experimenter view, allowing making an online monitoring of given responses by the experimenter (this made the task not purely computerized).

#### 3.6.3. Visual AVLT Task by the Touch-Screen Tablet

In the visual version of the task a total number of 10 target words were presented to the participant. Immediately after viewing these 10 target stimuli he/she was presented with the same 10 words randomized together with another set of 10 fillers (i.e., words not present in the targets lists) and asked to touch the screen each time he/she recognized an item belonging to the previously presented list. The same setting was used for the delayed part of the task, with the only exception that he/she was presented with the same 10 words randomized together with another set of 20 fillers, including the 10 fillers presented before. We chose 20 fillers for the delayed task because we tripled the number of the target words (10). This approach has been adopted in the original paper and pencil Rey auditory verbal test [24].

Prior to task administration, the experimenter handed the tablet to the participant, who was therefore actively required to use the testing apparatus while the experimenter would only passively control upon subject's performance.

Stimuli were presented as white words (with a font size of 36 points) at the center of a black screen and lasted for 3000 ms.; an ISI of 2000 ms occurred between one stimulus and the next one.

Accuracies were estimated by comparing each stimulus code (i.e., target or filler) to subject response and stored in a logfile.

### 3.7. Experimental Design for Testing Efficacy

A combination of one AVLT version (i.e., visual or verbal) and one ANT (i.e., touch or mouse) layout was administered to each subject, resulting in a factorial design in which (i) 19 subjects (10 males) performed the touch response layout of ANT and the other 19 subjects (9 males) performed the mouse response layout, and (ii) 19 out of 38 subjects (11 males) performed the visual version of AVLT while the remaining 18 subjects (9 males) performed the verbal version of AVLT.

Groups were statistically matched for age, education, and MMSE and no significant difference emerged when comparing the (i) mouse versus touch group (age *P* = 0.831; education *P* = 0.970; and MMSE *P* = 0.272) and the (ii) verbal versus visual group (age *P* = 0.970; education *P* = 0.487; and MMSE *P* = 0.344).

AVLT lists were randomly and evenly distributed across subjects for both verbal and visual versions of the task. Each subject was first asked to complete the immediate memory recall/recognition of AVLT and was tested 15 minutes later for the delayed, long-term retrieval component [24].

Two experimental runs of ANT (7 minutes each) were administered in between the two AVLT components as interference task, that is, a task critically involving neither the verbal nor the learning cognitive resources recruited during AVLT.

### 3.8. Ecology: Satisfaction Survey

After the administration of the tests, independently from the test (AVLT or ANT) and response layout (touch or mouse), participants were required to complete a satisfaction survey. The survey is a 16-item self-report questionnaire that uses a 5-point Likert scale (for the complete list of items of the questionnaire see Supplementary Materials available online at http://dx.doi.org/10.1155/2014/804723).

The survey measured the following: the participant frequency of use of touch-screen tablet and of mouse-controlled PC (items 1, 2, and 3), the participant qualitative perception of the familiarity with the touch-screen tablet (item 4), the participant qualitative perception of the comfortableness with the touch-screen tablet (items 5, 6, and 7), the participant qualitative perception of the testing environment (items 8 and 9), the participant fatigue of using sensory functions while interacting with the touch-screen tablet (items 10, 11, 12, 13, and 14), the participant fatigue of maintaining the concentration while interacting with the touch-screen tablet (item 15), and the participant time perception of the neuropsychological tests (item 16).

Items administration was customized depending on the experimental (i.e., different combinations of touch or nontouch versions of AVLT and ANT) setting of each participant.

## 4. Data Analysis

### 4.1. Testing Efficacy I: Executive Function Assessment

In order to assess possible differences between the two settings (touch-screen tablet versus mouse-control PC), we evaluated the recorded hardware uncertainties to control for specific setting effects (touch versus mouse) on stimulus presentation. Hardware uncertainties are provided by the presentation software giving information on how the hardware is managing stimuli presentation and if some hardware-based source of variability is altering the script management by the software. Specifically, for each Presentation event in the logfile (except for pause, resume, and quit events), presentation provides a time of occurrence *T* (ms) and an uncertainty *dT* (ms). These two numbers provide bounds on the time of occurrence of a presentation event. If Hardware uncertainties *dT* remain less than 0.6 ms the response script is running as expected (http://www.neurobs.com/, Presentation help). Metrics measured by hardware uncertainties refer to time responses of the device to the stimuli software presentation, independently from the subject response to stimuli presentation.

#### 4.1.1. Data Screening

Concerning the ANT, we considered the distributions of reaction times (RT) and accuracies. Specifically, with respect to reaction times (RT), any trial with recorded RT that fell two SDs above or below the calculated RT mean (RT ≥ mean + 2SD) (RT ≤ mean ± 2SD) was rejected.

With respect to accuracy each experimental condition with measured accuracy that fell under 80% was rejected.

#### 4.1.2. Statistical Analysis

Based on the selected datasets, we performed a 3 × 2 repeated measures ANCOVA with effect type (i.e., conflict effect, alerting effect, and orienting effect) as within-subjects factor with three levels.

A between-subjects factor was considered, namely, the participant group with two levels (mouse versus touch layout). Although groups were sampled with comparable values of MMSE, age, and education (see Section 3.7 *Experimental Design for Testing Efficacy* for groups comparisons statistics) these variables were included as covariates, in order to account for their potential influence on task performance. We assessed the main effect of group of participants, covariates, and exclusively interactions with the between-subjects factor (i.e., group of participants).

### 4.2. Testing Efficacy II: Memory Function Assessment

Concerning the verbal AVLT, vocal recordings were listened to and classified as correct or incorrect, while, for the visual AVLT, responses to target stimuli were stored in a logfile and successively coded as correct or incorrect.

Accuracies for the three immediate recall sessions (i.e., IR-1, IR-2, and IR-3) and the delayed recognition session (DR) were coded as percentages. Analyses of equality between verbal and visual AVLT were performed with a Mann-Whitney *U* test (nonparametric) for independent samples (i.e., according to AVLT verbal or visual condition) on the arcsine-transformed percentages of accuracy (for each recall) and the delayed session independently. Correlations between the MMSE scores and the arcsine-transformed percentages of correct responses for IR-1, IR-2, IR-3, and DR were then evaluated using Kendall's tau correlation coefficient split by AVLT verbal and AVLT visual conditions.

### 4.3. Ecology

Firstly, we performed one sample Wilcoxon signed rank test for each of the 16 items, measured by the survey administered to the participants, versus the middle level of perceived scale quality (3 with respect of a scale maximum of 5). Furthermore, we investigated the relationship between the level of preference of participants for the testing environment (i.e., item 7, using the touch-screen tablet with respect to an external device such as a mouse or a keyboard) and the perceived degree of easiness when using a touch-screen tablet (i.e., item 5) or the perceived degree of easiness when touching the screen (i.e., item 6).

## 5. Results

### 5.1. Testing Efficacy I: Executive Function Assessment

No anomaly linked to hardware management of stimuli presentation was detected when screening both mouse and touch logfiles.

#### 5.1.1. Data Screening

All subjects had an RT outlier percentage <30% on each experimental condition and accuracies were >80% for all participants; no subject was therefore excluded from the analyses.

#### 5.1.2. Statistical Analysis Results

Mean RTs for trial conditions for the calculation of each of the three effects are summarized in Table 3. The main effect of group of participants was found not to be significant (*F*(1,33) = 0.008, *P* = 0.929) revealing that performances are comparable in terms of overall RTs for the ANT with mouse or touch response layout. No significant main effects of covariates were found (MMSE *F*(1,33) = 0.049, *P* = 0.826; age *F*(1,33) = 1.221, *P* = 0.277; and education *F*(1,33) = 0.359, *P* = 0.553).

**Figure 2:**
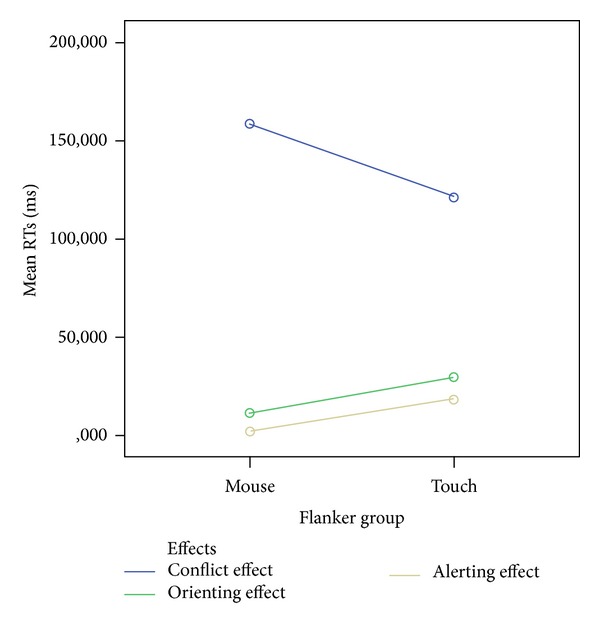
Plot of 2-way interaction group (mouse or touch) *X* effect (conflict effect, orienting effect, and alerting effect). Mean RTs (*y*-axis) for conflict Effect (Blue Line), orienting Effect (Green Line), and alerting Effect (Brown Line) are shown for mouse (leftmost dots) and touch (rightmost dots) performances (*x*-axis).

The interaction between group of participants and effects showed a trend toward significance (*F*(2,66) = 2.357, *P* = 0.116, Greenhouse-Geisser correction) and a plot of this 2-way interaction showed a reduced CE and an incremental AE and OE effect for the touch group (see Figure 2); pairwise, post hoc comparisons revealed a trend towards significance when considering CE differences between groups (mean difference, Touch − Mouse = −37.280; *P* = 0.170) in terms of a reduced CE for the touch, while the two groups differed to a lesser degree for the OE (Mean Difference, Touch − Mouse = 18,218; *P* = 0.232) and AE (Mean Difference, Touch − Mouse = 16,418; *P* = 0.261).

### 5.2. Testing Efficacy II: Memory Function Assessment

No significant differences were observed between verbal and visual recall/recognition performances for IR-1 (Mann-Whitney test; *P* = 0.365), IR-2 (Mann-Whitney test; *P* = 0.293), IR-3 (Mann-Whitney test; *P* = 0.694), and DR (Mann-Whitney test; *P* = 0.988).

For AVLT verbal, MMSE scores correlated with performance on IR-1, IR-2 immediate and with the delayed recall session; a trend towards correlation was found for the third immediate recall session.

For AVLT visual, MMSE scores showed a correlation trend with IR-1 and a significant correlation with IR-2, IR-3, and DR.

For AVLT verbal all immediate and delayed recall sessions significantly correlated with age.

For AVLT visual, recognition sessions did not significantly correlate with age.

For AVLT verbal, education scores significantly correlated with all the immediate and the delayed recall sessions. For AVLT visual, education scores did not correlate with immediate recall sessions while a significant correlation was found with education for the delayed recall session.  Table 4 summarizes correlations coefficients and statistical significance between each AVLT recall session and socio demographics (age and education) and cognitive index (MMSE) variables tested.

### 5.3. Ecology

Wilcoxon one sample signed rank test indicated that the middle percentage of quality ratings was significantly lower than 3 for item 1 (“how much do you use the tablet in your daily life?” *P* = 0.003), for item 4 (did you feel uncomfortable using the tablet?, *P* = 0.000), for item 10 (did you feel fatigued while handling the tablet?, *P* = 0.000), for item 12 (did you feel fatigued while touching the screen?, *P* = 0.000), for item 13 (did you feel fatigued while listening to vocal recordings?, *P* = 0.005), for item 14 (did you feel fatigued producing a vocal response?, *P* = 0.001), and for item 16 (did you feel the experiment had a too long duration?, *P* = 0.000), which is indicative of very low levels of uneasiness (i.e., item 4) or fatigue of using sensory functions (i.e., items 12, 13, and 14) and a perceived very long duration of test administration (i.e., item 16), notwithstanding a very low use of a touch-screen tablet in everyday life (i.e., item 1). The relationship between the preference for using the touch screen (i.e., item 7) and the perceived degree of easiness when using a tablet (i.e., item 5, Kendall's tau = 0.484, *P* = 0.009) or the perceived degree of easiness when touching the screen (i.e., item 6, Kendall's tau = 0.397, *P* = 0.025) were both significant (Kendall's tau = 0.83, *P* = 0.02) (see Supplementary Materials online for the complete items list).

## 6. Discussion

As general consideration, for the purpose of our work, we have chosen two representative tests to assess two main objectives.

We investigated the memory domain, given that the earlier cognitive symptoms reported in Alzheimer's disease, the most common form of dementia, involve memory [28]. Therefore, the majority of tools have focused mainly on this cognitive ability and have implemented tests tailored at investigating memory impairments, of which, the most clinically validated and commonly employed in a clinical setting is the AVLT test. Furthermore, memory impairments, in particular, are a cardinal feature of the majority of dementia syndromes.

We investigated the executive function domain by the use of the ANT, as a useful test for assessing differences between responses, given that it encompasses three different effects relying on three different cognitive mechanisms (i.e., conflict effect, alerting effect, and orienting effect) [29].

The first aim of the present study was to evaluate the testing efficacy of a computerized neuropsychological assessment when implemented on a touch-screen device.

To this aim, we created and tested two different experimental settings in terms of response layout; specifically, two identical versions of an experimentally validated attentional task (i.e., ANT; [22, 23]), differing only in terms of response modality were implemented: one version of the task required subjects to give a response with a mouse device while the other one by using a touch screen. This was made in order to directly compare the testing efficacy of a psychological test (in our case the evaluation of executive function) when administered by a touch-screen device with respect to a more conventional mouse-control PC.

Comparisons of reaction times between subjects using touch screen or mouse and of their test performance revealed no significant overall differences, suggesting that touch screen and mouse can be equally chosen as response devices, since they grant the same experimental outcome. These findings strengthen the results highlighted by Sears and Shneiderman [30] who, although under different experimental conditions, compared touch-screen response layout versus mouse response layout. Their results suggested substantial comparability between these two input devices. Our finding, consistently with results from other authors, is of particular interest, given that the touch-screen technology is currently widely spreading, also among the elderly population. Touch-screen tablets are innovative technological solutions which are emerging also as devices for healthcare intervention. Healthcare services are indeed progressively showing an increasing interest in translating services into touch-screen based environment [31, 32]. From this perspective it is an important topic to test if such devices can guarantee the same testing efficacy of more conventional and extensively validated devices, as mouse-control PC, which are still today used in clinical environment to administer neuropsychological tests. Our work, despite being validated on a limited number of subjects, suggests that this technological solution is feasible for test administration.

The second aim of our work was to evaluate the testing efficacy of a computerized test (in a representative case of memory function assessment) when administered by the touch-screen tablet with respect to two different experimental settings in terms of stimuli presentation.

To this aim, we created and tested two different versions of AVLT, a widely standardized and validated neuropsychological test: (i) a visual version, replicating the visual porting of this task which is currently used by a set of different digital neuropsychological training batteries [19, 33]: in this versions stimuli were presented visually and subjects were asked to recognize memorized stimuli among other nontarget stimuli and (ii) a verbal version, replicating the “classical” administration of the task in the clinical context: in this version stimuli were presented verbally and subjects were asked to freely recall all the memorized target stimuli. This was done with the purpose to assess the effects of the stimuli presentation modality on the efficacy of test (in our case the evaluation of memory function).

Our results for the verbal version of AVLT showed significant correlations between MMSE scores and performance on IR-1, IR-2 immediate and with the delayed recall session. A trend towards significance was found for the third immediate recall session (IR-3). Similarly, results for the visual version of AVLT showed a correlation trend between MMSE scores and performance with IR-1 and a significant correlation with IR-2, IR-3 and with the delayed recall session. For AVLT verbal, all immediate and delayed recall sessions showed an inverse and significant correlation with age (i.e., lower scores of age correspond to higher values of recall performance); education scores significantly and positively correlated with all immediate and the delayed recall sessions (i.e., higher values of education correspond to higher values of recall performance). For AVLT visual, a significant and direct correlation with recognition performance scores was found only with education for the delayed recall session and no significant correlations were found between age and performance on both immediate and delayed recognitions (see Table 4 for detailed results).

Overall these results suggest that both implementations of the test (i.e., visual and verbal) are affordable measures of the general cognitive status, directly correlated with a measure of general cognitive status assessment (i.e., MMSE); from this point of view they can be both considered affordable tools for a broad cognitive assessment. In spite of this only the verbal version of the task showed a correlation with the sociodemographical data of our sample (i.e., an inverse correlation with age and direct correlation with education).

It should be in fact recognized that we have not compared verbal with “pure” visual stimuli presentation, since filler words were presented during the task: under this light the two tasks share some common features but differ for others in terms of both experimental setting and underlying cognitive processes.

In fact the verbal version requires an active retrieval from memory of the presented stimuli, while the visual version requires the recognition and discrimination of the memorized target stimuli among other nontarget stimuli. From this point of view they require the subject to use different strategies to be solved and thus may involve different brain networks (e.g., [34]). For example from the memory point of view the visual modality could be easier since the subject is provided with a cue (namely, the target stimulus is directly presented to the subject) but at the same time it requires the inhibition of distracters and may be more difficult in terms of executive functioning cognitive load (namely, the subject has to discriminate the target stimulus among other nontarget words).

Another crucial aspect concerning the introduction of new technological solutions in the everyday life is the degree of ecology and the level of preference of the computerized assessment modalities regarding the administration of the neuropsychological tests. Although some authors found significant draw backs to touch screens in the elderly [35] others (e.g., [31]) reported that touch-screen devices are ideal instruments for assessing populations with low technological familiarity, such as elders and patients. Our results on elderly and healthy participants confirm this finding, considering that our subjects felt comfortable using the touch-screen device and did not experience unease or fatigue feelings while performing the tests. Crucially, all subjects possessed low familiarity with such devices and, in some cases, it was their first experience of physical interaction with a touch-screen tablet.

Given the performance comparability between responses using mouse and touch, it is important to introduce some considerations for future evaluation regarding whether (and under which circumstances) it is preferable to choose one or the other response layout.

While no main effect of group was highlighted, our analysis revealed that subjects performing the ANT task using the touch response layout showed a tendency towards an advantage for all three effects accounted by the task and, namely, a trend towards a significant reduction of conflict effect and slightly larger alerting and orienting effects (see Figure 2 and Table 3 for details).

However, it must be acknowledged that RTs from which the effects are derived showed the same pattern for both the mouse and the touch layout; namely, for the conflict effect RTs are the longer with respect to congruent ones while for alerting effect RTs measured following the double cue were shorter with respect to when no cue was presented, and for orienting effect responses after a spatial cue were faster with respect to a cue presented centrally. This means that there is no difference in terms of performance for all ANT conditions. However, when assessing specific cognitive processes measured through the difference between RTs it appears that the touch device may provide some benefits mainly on cognitive control, enhancing performance on the more demanding trial type. Namely, conflict effect is calculated by subtracting RTs belonging to congruent trials (flanking arrows pointing in the same direction of the central target arrow) from RTs belonging to incongruent trials (flanking arrows pointing in the opposite direction of the central target arrow). To this extent lower values of conflict effect indicate a higher performance on cognitive conflict resolution.

This finding becomes of particular interest when considering the kind of trial associated to this reduction in the effect size. Incongruent trials require the resolution of a cognitive conflict to be solved and they are known to be the slowest ANT experimental conditions in terms of response speed [22].

Forlines et al. [36] showed that tasks requiring the use of two hands (bimanual tasks, as the ANT) are better performed when using touch-screen devices with respect to mouse-control PC. To a similar extent Rogers et al. [37] found that touch-screen devices are particularly suitable for response collection when compared to another indirect response device (namely, a rotary encoder).

Given our results, it is important to acknowledge this proposed dichotomy between direct (the touch-screen, in our case) and indirect response devices. When using a direct input device, the distance between the subject (his/her fingers) and the causal effect he/she carries on the environment modification (touching stimuli on the screen, as required by the task) is reduced. Touch-screen devices, in this framework, lead a virtual environment to a more tangible and ecological dimension. One possible consequence of such phenomenon could be an increase in self-commitment or in self-perceived efficacy towards the task, and this could lead to an enhancement by establishing a direct link between the subject and the task reality. In other words, a different perception of the self-commitment could be associated with responses given with direct input devices, shifting the task environment perception into a more concrete entity on which the subject acts as a physical agent. Thus, critically, the subject involvement into the task could have been enhanced.

Under this light one would expect to observe a greater effect for those trials requiring a greater cognitive demand (i.e., incongruent trials). A greater involvement could translate into greater resources dedicated to task solution. To this extent, cognitively simpler trials (i.e., congruent trials) would benefit less, since they do need less work to be solved; on the contrary, trials requiring greater cognitive effort to be solved, such as incongruent trials, would greatly benefit from such resource availability.

Although this scenario is suggestive, some dedicated experimental investigation is needed to shed light on the cognitive basis of this behaviorally observed phenomenon.

These evidences, taken together with results on the ease of their use highlighted by the survey, indicate touch-screen devices as an ecological and suitable tool for the computerized administration of neuropsychological tests. Furthermore, other authors [35] showed that alternative response input devices, such as a light pen or touch screen are highly intuitive, and have the advantage of bypassing the keyboard. They demonstrate how these devices allow subjects to focus their attention directly on the video display terminal and not have to shift their attention from the monitor to the keyboard to locate a response key. Nevertheless, light pens and touch screens also have their disadvantages. They require the subject to hold his or her arm in an “up” position and move it along the screen. This can produce fatigue and some variation in reaction time.

It should be noted that the computerized assessment does not represent an alternative to the clinical setting. However, it can contribute in a significant manner to the traditional evaluation. Nevertheless, there is a need to further detail some aspects of our investigation: (i) in order to increase the inferential power and experimental validity of our findings, the tests will need to be administered to a larger number of participants; (ii) among these participants, specific cognitively-impaired populations and physically-impaired populations (e.g., those subjects with motor function deficit from a brain injury that could affect the test performance) will need to be tested in order to assess if these instruments can be a valid and accessible tools in the clinical context, and, (iii) most importantly, a dedicated version of cognitive domain-specific tests will need to be implemented and case-wise tested in order to detail whether, and to which extent, they can be a valid alternative to more conventional pc-based and/or pencil and paper testing approaches.

## 7. Conclusions and Future Perspectives

This work provides new data on the experimental feasibility and clinical ecology of computerized neuropsychological assessment by addressing the impact of the implementation of different user interfaces and different stimuli presentation modality.

In order to set up an innovative computerized testing environment, while keeping it feasible and ecological, it is fundamental to detail how this conversion process impacts the experimental and clinical neuropsychological settings. Although limited on approximately 40 healthy subjects and experimented only on representative, not exhaustive, neuropsychological tests (on memory and attention functions) our evidences suggest that touch-screen devices can be considered for the computerized administration of neuropsychological tests.

## Supplementary Material

Supplementary Materials of the paper “Computerized Neuropsychological Assessment in Aging: Testing Efficacy and Clinical Ecology of Different Interfaces” include a self-reported questionnaire administrated to all participants to the study. The questionnaire included 16 items measuring the following participant features: the participant frequency of use of touch-screen tablet and of mouse-controlled PC (items 1, 2, and 3), the participant qualitative perception of the familiarity with the touch-screen tablet (item 4), the participant qualitative perception of the comfortableness with the touch-screen tablet (items 5, 6, and 7),the participant qualitative perception of the testing environment (items 8 and 9), the participant fatigue of using sensory functions while interacting with the touch-screen tablet (items 10, 11, 12, 13, and 14), the participant fatigue of maintaining the concentration while interacting with the touch-screen tablet (item 15), and the participant time perception of the neuropsychological tests (item 16). Participants were asked to rate each item on a 5 points Likert scale. Scores ranged from 1 (very poor) to 5 (very much).

## Figures and Tables

**Table 1:** Features of computerized cognitive batteries cited in Section 2. Hardware, input modality, and context of use information are provided.

Battery name	Hardware used	Input modality	Use
CNS vital signs	PC/laptop	Keyboard	Research use
CogState	PC/laptop/tablet	Keyboard	Research use
	(Web based)		
Neurotrax	PC/laptop (web based)	Keyboard/mouse	Clinical use (AACN consensus)
IntegNeuro	PC/laptop	Keyboard	Research use
Touch panel dementia assessment scale	PC/laptop	Touch screen	Research use
CADi	iPad	Touch screen	Research use
CANTAB mobile	iPad	Touch screen	Research use

**Table 2:** Age, education, and MMSE scores are provided for the whole (*N* = 38) group (left column). Independent sample Student's *t*-tests values for these variables are shown for the AVLT (verbal versus visual) input modality groups (center column) and for the ANT (touch versus mouse) response modality groups.

	Overall group *N* = 38 20 M/18 F	AVLT groups *N* = 38 visual group (11 M/8 F) verbal group (9 M/10 F) Student's *t*-test (*P* values)	ANT groups *N* = 38 touch group (10 M/9 F) mouse group (9 M/10 F) Student's *t*-test (*P* values)
Age	mean 64.474	0.970	0.831
	st dev 8.462		
	range 53 : 87		

Education	mean 11.263	0.487	0.970
	st dev 4.131		
	range 5 : 19		

MMSE	mean 28.91	0.344	0.272
	st dev 0.67		
	range 28 : 30		

**Table 3:** Mean RTs (along with SD) for each experimental condition used to calculate effects of ANT are shown in the table for both touch (upper panel) and mouse (lower panel) versions of the task. Congruent trials are subtracted from incongruent trials in order to calculate conflict effect, double cue trials are subtracted from no cue trials in order to calculate the alerting effect, and spatial cue trials are subtracted from central cue trials in order to calculate the orienting effect.

Trial type	Mean	SD	Trial type	Mean	SD	Effect	Mean	SD
Touch layout								
Incongruent	1016.229	122.973	Congruent	890.436	125.071	Conflict effect	125.793	61.692
No cue	936.42	122.244	Double cue	914.954	127.472	Alerting effect	21.466	49.6
Central cue	932.806	117.579	Spatial cue	908.124	129.387	Orienting effect	24.682	34.646

Mouse layout								
Incongruent	941.746	181.163	Congruent	786.627	109.758	Conflict effect	155.118	89.207
No cue	833.05	125.772	Double cue	828.475	135.419	Alerting effect	4.57	34.988
Central cue	830.022	129.443	Spatial cue	820.197	140.739	Orienting effect	9.825	54.498

**Table 4:** Results show correlations coefficients (upper values in cells) and statistical significance (lower values in cells) between each AVLT recall session (i.e., IR-1, IR-2, IR-3, and DR) and sociodemographics (age and education) and cognitive index (MMSE) variables tested. The left most panel of table shows statistics belonging to the group tested with verbal version of the AVLT while the rightmost panel shows statistics belonging to the group tested with the visual version of the task.

	Verbal				Visual			
	IR-1 (*τ*)	IR-2 (*τ*)	IR-3 (*τ*)	DR (*τ*)	IR-1 (*τ*)	IR-2 (*τ*)	IR-3 (*τ*)	DR (*τ*)
MMSE	0.633	0.492	0.373	0.406	0.246	0.579	0.451	0.407
	(*P* = 0.001∗∗)	(*P* = 0.013∗)	(*P* = 0.040∗)	(*P* = 0.069)	(*P* = 0.195)	(*P* = 0.003∗∗)	(*P* = 0.020∗)	(*P* = 0.035∗)

Age	−0.54	−0.479	−0.434	−0.353	−0.137	−0.19	0.007	−0.216
	(*P* = 0.031∗∗)	(*P* = 0.008∗∗)	(*P* = 0.050∗)	(*P* = 0.240)	(*P* = 0.448)	(*P* = 0.303)	(*P* = 0.970)	(*P* = 0.240)

Education	0.448	0.527	0.408	0.404	0.183	0.25	0.164	0.38
	(*P* = 0.017∗)	(*P* = 0.006∗∗)	(*P* = 0.039∗)	(*P* = 0.034)	(*P* = 0.332)	(*P* = 0.197)	(*P* = 0.409)	(*P* = 0.049∗)

(*τ* = Kendall's tau. ∗Correlation is significant at the 0.05 level, 2-tailed).

(∗∗Correlation is significant at the 0.01 level, 2-tailed).
