# Supplementary material for: Computerized Neuropsychological Assessment in Aging: Testing Efficacy and Clinical Ecology of Different Interfaces
Source: Comput Math Methods Med. 2014 Jul 24;2014:804723. doi: 10.1155/2014/804723 (PMC4131509; doi:10.1155/2014/804723)
Supplement: Supplementary file 1 — Supplementary Materials of the paper “Computerized Neuropsychological Assessment in Aging: Testing Efficacy and Clinical Ecology of Different Interfaces” include a self-reported questionnaire administrated to all participants to the study. The questionnaire included 16 items measuring the following participant features: the participant frequency of use of touch-screen tablet and of mouse-controlled PC (items 1, 2, and 3), the participant qualitative perception of the familiarity with the touch-screen tablet (item 4), the participant qualitative perception of the comfortableness with the touch-screen tablet (items 5, 6, and 7),the participant qualitative perception of the testing environment (items 8 and 9), the participant fatigue of using sensory functions while interacting with the touch-screen tablet (items 10, 11, 12, 13, and 14), the participant fatigue of maintaining the concentration while interacting with the touch-screen tablet (item 15), and the participant time perception of the neuropsychological tests (item 16). Participants were asked to rate each item on a 5 points Likert scale. Scores ranged from 1 (very poor) to 5 (very much). [file 804723.f1.pdf]

Supplementary Materials

1 Satisfaction Questionnaire

This is a self-reported questionnaire; participants were asked to rate each item on 5 point Likert scale. Scores ranged from 1 (very poor) to 5 (very much).

Item 1. How much do you use the tablet in your daily life?

Item 2. How much do you use the computer in your daily life?

Item 3. How often do you use a tablet to navigate the web?

Item 4. Did you feel uncomfortable using the tablet?

Item 5. How much did you feel at ease using the tablet?

Item 6. How much did you feel at ease touching the screen?

Item 7. How much did you prefer to touch the screen, instead of using an external device (mouse/keyboard) to interact with the task?

Item 8. How much should you have preferred to perform the test using a standard pc instead of a tablet device?

Item 9. How much should you have preferred to have a physical person administering the tests?

Item 10. Did you feel fatigued while handling the tablet?

Item 11. Did you feel fatigued while having to pay attention to the screen?

Item 12. Did you feel fatigued while touching the screen?

Item 13. Did you feel fatigued while listening to vocal recordings?

Item 14. Did you feel fatigued producing a vocal response?

Item 15. Did you feel fatigued to sustain your attention?

Item 16. Did you feel the experiment had a too long duration?
